# Applications of Converged Various Forces for Detection of Biomolecules and Novelty of Dielectrophoretic Force in the Applications

**DOI:** 10.3390/s20113242

**Published:** 2020-06-07

**Authors:** Seungjun Lee, Seong Min Roh, Eunji Lee, Yejin Park, Byung Chul Lee, Youngeun Kwon, Hye Jin Kim, Jinsik Kim

**Affiliations:** 1 Department of Medical Biotechnology, Dongguk University, Seoul 04620, Korea; zldeja75@dongguk.edu (S.L.); rsm0321@dgu.ac.kr (S.M.R.); leeej517@dgu.ac.kr (E.L.); jinne1021@dongguk.edu (Y.P.); ykwon@dgu.ac.kr (Y.K.); 2 Center for BioMicrosystems, Korea Institute of Science and Technology (KIST), Seoul 02792, Korea; bclee@kist.re.kr; 3 Department of Clinical Pharmacology, Kyung Hee University, Seoul 02447, Korea

**Keywords:** biomolecule separation, dielectrophoresis, electrophoresis, acoustophoretic, electroosmotic flow force, magnetophoretic force, electrokinetic force, hydrodynamic force, optical trapping force

## Abstract

Since separation of target biomolecules is a crucial step for highly sensitive and selective detection of biomolecules, hence, various technologies have been applied to separate biomolecules, such as deoxyribonucleic acid (DNA), protein, exosome, virus, etc. Among the various technologies, dielectrophoresis (DEP) has the significant advantage that the force can provide two different types of forces, attractive and repulsive DEP force, through simple adjustment in frequency or structure of microfluidic chips. Therefore, in this review, we focused on separation technologies based on DEP force and classified various separation technologies. First, the importance of biomolecules, general separation methods and various forces including DEP, electrophoresis (EP), electrothermal flow (ETF), electroosmosis (EO), magnetophoresis, acoustophoresis (ACP), hydrodynamic, etc., was described. Then, separating technologies applying only a single DEP force and dual force, moreover, applying other forces simultaneously with DEP force were categorized. In addition, advanced technologies applying more than two different kinds of forces, namely complex force, were introduced. Overall, we critically reviewed the state-of-the-art of converged various forces for detection of biomolecules with novelty of DEP.

## 1. Introduction

In biomedical applications, detecting and analyzing specific biomolecules is important for successful investigation of physiological mechanisms by discovering new roles of biomolecules and for accurately diagnosing of various diseases. However, most of biomolecules are present in complex biological buffers composed of tens or thousands of components, such as blood. Therefore, a technique, such as sorting, isolation or filtration for separating various biomolecules, is required in order for accurate detection and analysis a specific biomolecule.

Among various separation techniques, physical filtration of samples, such as ultracentrifugation, has been conventionally used to remove other unnecessary components [1,2]. The physical methods are no doubt the most powerful for removing unnecessary analytes from a sample, however, those methods could also reduce the amount of target analytes or damage target molecules during the separation procedures [3]. Besides, when detecting target analytes with ultra-low concentration, separation of the target analytes through physical methods could be a serious defect that prevents them from being detected. In spite of the defect, most of the recent research focuses on target analytes in ultra-low concentrations ranging from fg/mL to pg/mL [4]. Thus, various methods have been developed to accurately separate the target molecules by enlarging the concentration of molecules with immunoaffinity, ionic strength or electrical force. There are representative methods for separating various biomolecules such as DNA, protein or exosomes: polymerase chain reaction (PCR) [5], electrophoresis (EP) [6], magnetic force with bead [7], dielectrophoresis (DEP) [4], etc. These methods improve the detection probability of target biomolecules by increasing the concentration of target biomolecules other than non-target biomolecules. 

Nowadays, many kinds of target biomolecules, such as exosomes, which can inform the status of health of living things are varied, including conventional analysis of deoxyribonucleic acid (DNA) or protein. Therefore, more complex separation techniques using physical and biochemical reactions are spotlighted compared to conventional separation methods. For this reason, various forces should be applied to detect the target molecules by sorting, isolation or filtration. As a result, separation techniques, including several processes with physical, electrical or biochemical methods, have become more complicated.

These complex technologies using a variety of processes can be easily implemented by reducing several steps based on a microfluidic chip technology [8], called lab-on-a-chip; especially, a flow to reduce processes in inducing complex and DEP force could be a splendid candidate as a tool for separating. Because DEP force can produce a different direction of force—positive and negative DEP (p-DEP and n-DEP) force—just by changing the frequency to make different types of DEP forces in a microfluidic chip with the same electrode and structure [9]. The p-DEP is attractive force to accomplish attachment of biomolecules to electrode. On the contrary, the n-DEP is repulsive force to perform the detachment of biomolecule from electrode. From the characteristics of DEP force, various functions such as trapping or extrusion in microfluidic chip can be carried out with adjustment of the direction of DEP force at same apparatus. It is also notable characteristics to elicit various roles for sorting, isolation or filtration when used in combination with other types of forces. Utilization of DEP forces in developing convergence of various forces could provide more complex functions for more sensitive detection without additional apparatus or steps.

In this paper, the reviewed results for various techniques of detecting and analyzing through separation of biomolecules, such as DNA, protein, exosomes, and cells, were introduced. Definitions of each force (DEP, EP, electrothermal flow (ETF), electroosmosis (EO), magnetophoretic force, hydrodynamic force, and optical trapping force), which can be utilized as a single unit force in the convergence, were also clearly organized, with properties of biomolecules which can affect the single force. Depending on the complexity of the utilized forces, the separating techniques of biomolecules were categorized into a single DEP force, two different forces, and more complex forces usage to show the variable applications of DEP force in convergence of various forces.

## 2. Classifying Multifarious Biomolecules by Representative Separating Methods

### 2.1. DNA

DNA is a nucleic acid that contains the information needed to encode proteins and other cellular components. Therefore, it is possible to predict and diagnose various DNA-related syndromes and diseases by analyzing chromosomal disorders, such as deletion, replication, translocation, and detecting disease-related tumor suppressing DNA [8,10]. To detect and analyze, an effective method is required for separating small amounts of DNA present in the sample, such as physical centrifugation, PCR, EP, etc. Centrifugation is the most classic method of separating DNA from pellets obtained after lysis of biomolecules, such as cells, exosomes, and proteins. Therefore, various studies have been reported on centrifugation-based DNA separation methods, such as optimal centrifugal speed, precipitation rate of DNA with buffer, and recovery efficiency of DNA after centrifugation [11,12]. PCR is the most representative method used to selectively amplify the target fragments of DNA in vitro and enables high efficiency separation and detection of DNA. Using PCR, Jeff et al. separated approximately 53.5 ± 10.7 ng/μL of DNA within 30 min from *Xylella fastidiosa* bacteria [13]. In addition to that research information, there were various methods to separate and detect the DNA, such as EP-based and nanoparticles-based separation methods [5,10]. Min et al. and Türkcan et al. used the dimercaptosuccinic acid (DMSA) coated magnetic nanoparticles (MNP) and silanized polymeric nanoparticles, demonstrating a separation efficiency of approximately 86.16% and detection limit of about 50 bp, respectively.

### 2.2. Protein

Proteins are organic compounds composed of amino acids and play an important role in understanding metabolism and body physiology and diagnosing diseases. Thus, various attempts have been made to develop innovative protein separation methods with high reliability and sensitivity. Same with the methods to separate the DNA, protein is separated through physical centrifugation and size exclusion, immune affinity-based methods, EP, etc. Among the separation methods, centrifugation is widely used because of its simplicity and high separation efficiency. However, the methods can cause loss of protein concentration by aggregated protein pellets after centrifugation, as well as protein denaturation [14]. Immune affinity-based separation methods include systematic evolution of ligands by exponential enrichment (SELEX), PCR, etc. Zirath et al. separated a-fetoprotein (AFP) and Interleukin-8 (IL-8) with nanoparticles coated with antibody in microfluidic channels and demonstrated a sensitivity of 0.2 pg/mL in undiluted calf serum [15]. Lisi et al. separated the tau protein, which is strongly related to Alzheimer’s disease, using fluorophore-tagged aptamer and achieved a detection limit of 1.86 ± 0.19 ng/μL within 30 min [16]. In addition, similar to the physical separation methods, EP is also widely used for protein separation because of its simplicity and high efficiency [6]. 

### 2.3. Exosome

Exosomes are a microvesicle (MV) released from cells and contain various components, such as nucleic acids, proteins, lipids, amino acids and metabolites [2,17]. Therefore, they provide a variety of information about the state of a cell or tissue via intracellular and intercellular communication, which suggests a possibility for disease diagnosis and prediction by detecting exosomes [18,19]. These possibilities of exosome detection have led to the development of various physical and immune affinity-based methods to separate exosomes effectively. First, physical methods, such as centrifugation, precipitation, and size exclusion chromatography (SEC), are common methods used to separate exosomes [20,21,22]. Among these physical methods, ultracentrifugation is the simplest method that has been utilized until now, but the amounts of unwounded exosomes (recovery <25%) are very few and this technique is time-consuming (4–5 h), which is ineffective [20]. In addition, it is difficult to expect high recovery of exosomes and inappropriate to apply practical diagnosis and treatment because an additional separation process is required for analysis [22]. Therefore, in order to reduce the separation time of exosomes and increase the separation efficiency, many researchers attempted to separate exosomes using microfluidic channels [23,24]. The immune affinity-based methods are separated exosomes by using membrane surface marker existing on the surface of the exosome [25]. Chen et al. introduced an immune affinity-based method to separate exosomes using the anti-CD36 antibody, which is a specific antibody of exosomes and the most common surface protein in the exosome [26]. Through the methods, relatively small amounts of exosomes are separated under an hour. Son et al. detected exosomes derived from the cancel cell using an immune affinity-based method and fluorescence bead [27], and Fang et al. detected exosomes of Michigan Cancer Foundation-7 (MCF-7), a kind of cancer cell line, using a magnetic bead, which conjugated with CD 36 antibody [18]. In addition to the mentioned methods, acoustic wave-based method and viscoelastic-based method are used to separate exosomes [28,29].

### 2.4. Virus and Cell

Viruses containing genetic material and protein coating cause not only common human diseases, including colds, influenza, chickenpox and cold sores, but also serious diseases like Ebola and Acquired Immune Deficiency Syndrome (AIDS). Moreover, pathogenic bacteria, such as Yersinia pestis and methicillin-resistant Staphylococcus aureus, cause epidemics and lead to death [30,31]. Therefore, analyzing and detecting viruses and bacteria are important for the diagnosis of acute infectious disease and research of antibacterial and vaccine development. The most commonly used methods to separate and detect the virus and bacteria were immune affinity-based methods, such as PCR, immunoblotting, immunoprecipitation, and enzyme-linked immunosorbent assay (ELISA) [32,33,34,35]. Physical centrifugation and mass spectrometry also were widely used for viruses and bacteria separation [36].

## 3. Manifold Forces for Detecting of Biomolecules

We have briefly outlined not only the importance of detecting DNA, protein and exosomes, but representative methods for separating biomolecules based on the simple physical centrifuge and immunoaffinity in the previous section. However, these methods require at least 1 mL of analytical sample and bulky separation systems, and to improve these drawbacks, researchers have introduced a microfluidic channel to the separation of biomolecules. In microfluidic channels, biomolecules can be separated by forces arising depending on various properties of biomolecules such as size, density, permittivity, surface charge and permeability, not to mention the separation based on the immunoaffinity (Table 1).

In addition, these forces for separating biomolecules can be applied complexly in the microfluidic channels, enhancing the separation efficiency of biomolecules, and accordingly, the techniques of separating biomolecules using two or more different forces simultaneously, based on microfluidic channels, have recently been spotlighted. Before explaining such separation methods’ multiple forces, in the following sections, we have focused on classifying and describing the various separating forces due to the properties of biomolecules. Therefore, before explaining such methods for biomolecules separation using multiple force, we firstly have described and classified various separating forces caused by the properties of biomolecules.

### 3.1. Dielectrophoretic Force

When polarizable nano/micro particles are exposed in a non-uniform electric field, a difference in permittivity inside and outside the particles induces a dipole momentum of particle. The momentum causes the particles to move into the low- or high-density gradient region of the electric field; these behaviors are defined as DEP and the force causing the particles movement is known as DEP force [4]. The intensity of DEP force depends mainly on a strength of electric field (E) and a radius of particles (r) as follows [39]:

$$F_{DEP}=2\pi r^{3}\varepsilon_{m}Re(f_{cm})\nabla {\left|E\right|}^{2}$$

$$f_{cm}=\frac{\varepsilon_{p}^{*}-\varepsilon_{m}^{*}}{\varepsilon_{p}^{*}+2\varepsilon_{m}^{*}}$$

where $Re(f_{cm})$ is the real part of Clausius–Mossotti factor, $\varepsilon_{p}^{*}$ and $\varepsilon_{m}^{*}$ correspond the complex permittivity of particle and medium, respectively. The complex permittivity is expressed as follows:

$$\varepsilon^{*}=\varepsilon -\left(j\frac{\sigma }{\omega }\right)$$

where ε represents the real permittivity, σ is the conductivity, and ω corresponds the angular frequency of applied electric field. Thus, $\varepsilon_{p}^{*}$ and $\varepsilon_{m}^{*}$ change depending on the frequency, which changes the $f_{cm}$ value from −0.5 to 1 and decides the type of DEP force: if the value of the real part of $f_{cm}$ has a positive, the particle is attracted to a region with a high electric field density, which is called a p-DEP. In the opposite case, if the value of the real part of $f_{cm}$ has a negative, n-DEP force occurs and the particle is pushed to a region having a low electric field density. When DEP force is generated by direct current (DC) voltage, $Re(f_{cm})$ is specified as follows [39]:

$$f_{cm}=\frac{\sigma_{p}^{*}-\sigma_{m}^{*}}{\sigma_{p}^{*}+2\sigma_{m}^{*}}$$

where $\sigma_{p}^{*}$ and $\sigma_{m}^{*}$ correspond the complex conductivity of particle and medium, respectively. In summary, DEP force can trap, sort and isolate the particle according to (1) characteristics of the particle, such as size, shape, surface electrical properties, (2) the suspending medium’s properties and (3) applied electric field, which produces the non-uniform field gradient. 

### 3.2. Electrophoretic Force

Similar to the fact that polarizable particles are affected by DEP force, electrically charged particles are affected by the force under the electric field. When charged particles are in an electric field, the particles move towards to the electric field according to their surface charge by the Coulomb force; that is, the particles with a positive charge move along the direction of the electric field, and in the opposite case, particle movement in the opposite direction of the electric field occur. This phenomenon is defined as EP, and the force causing particle movement by EP is estimated as follows [40]:

$$F_{EP}=6\pi \eta r\mu_{EP}E$$

where η is the viscosity of the medium and $\mu_{EP}$ is electrophoretic mobility. As shown in the equation above, the EP force moves charged particles according to the intensity of the electric field and the charge density of particles.

### 3.3. Electrothermal Flow Force

When an electric field is applied to the medium to induce EP and DEP force, current flow is applied through the medium to produce joule heating. The joule heating induces a gradient of the permittivity and conductivity of the medium, creating an ETF that hinders the movement of particles [28]. The force generated by the ETF is defined as the electrothermal force and can be applied to move and separate particles, and force is expressed as follows [39]:

$$F_{T}=\frac{k_{B}\Delta T}{2r}\propto \sigma V^{2}$$

where σ represent the conductivities of the fluid and $k_{B}$ is the Boltzmann constant, ΔT and r are temperature and radius of the particle, respectively. Generally, electrothermal force is mostly significant under low frequency [41].

### 3.4. Electroosmotic Flow Force

Electroosmosis is the motion of liquid induced by an applied electric field along the interface of liquid and solid surface by an electric double layer (EDL) on the interface [42]. When the electrolyte is injected into the microfluidic channel which is electrically negative, ions are adsorbed onto the surface of the channel, forming EDL at the interface between the surface and the electrolyte fluid. If an electric field is applied to this channel, the motion of charge in the EDL is hindered by the electrostatic force. As a result, wall-slipping flow is induced at the EDL, making ununiform velocity profile across the channel, namely, electroosmotic flow (EOF) occurs. A velocity of the particles in EOF is calculated with the Helmholtz–Smoluchowski fluid “slip” formula as follows [43]:

$$\mu_{EOF}=-\frac{\varepsilon_{m}\zeta E}{\eta }$$

where ζ corresponds to a potential drop across the diffusion layer, namely zeta potential. EO affects the electrical conductivity of fluid and applied frequency: Relatively high conductivity decreases the thickness of the EDL, and relatively high frequency beyond 100 kHz or relatively low frequency makes EDL unable to be formed or makes net flow be not generated [42,44].

### 3.5. Magnetophoretic Force

When a magnetic field is applied, magnetically permeable particles can be isolated or trapped by force in a certain direction, and force is defined as magnetophoretic force and can be expressed as follows [7]:

$$F_{M}=\mu_{0}\left(\left(m_{p}-m_{f}\right)\cdot \nabla \right)H_{a}$$

where $\mu_{0}$ represents the permeability of free space, ∇ and $H_{a}$ are the Hamilton operator and an intensity of applied magnetic field, respectively. Additionally, $m_{p}$ corresponds to the magnetic moment of the particle and $m_{f}$ is the effective moment of the liquid surrounding the particle.

### 3.6. Acoustophoretic Force

Manipulation of particle position and sorting by using acoustic waves is defined as acoustophoresis (ACP) force [45]. To generate ACP force, standing wave is the most commonly used and the acoustophoretic or acoustic radiation force exerted on the particle by standing wave is calculated as follows [46]:

$$F_{r}=-\left(\frac{\pi p_{0}^{2}V_{p}\beta_{m}}{2\lambda }\right)\phi \left(\beta ,\rho \right)\sin\left(\frac{4\pi x}{\lambda }\right)$$

$$\phi \left(\beta ,\rho \right)=\frac{5\rho_{p}-\rho_{m}}{2\rho_{p}+\rho_{m}}-\frac{\beta_{p}}{\beta_{m}}$$

where $p_{0}$ and $V_{p}$ represent the acoustic pressure and the volume of the particle, $\beta_{p}$ and $\beta_{m}$ are the compressibility of the particle and medium, $\rho_{p}$ and $\rho_{m}$ correspond to the density of the particle and medium, and λ and ϕ represent the wavelength of the acoustic waves and acoustic contrast factor, respectively. Acoustic waves are usually produced by transducers made of piezoelectric materials converting electrical polarization into physical deformation changes and vice versa. 

### 3.7. Hydrodynamic Force

Fluid properties, such as flow rate, viscosity, density, and the various structures of microfluidic channels, produce a variety of fluid flows affecting the motion of particles in the fluid [47]. The forces affecting particle motion based on various fluid flows are defined as hydrodynamic force, and there are various particle separation mechanisms based on those forces such as deterministic lateral displacement (DLD), pinched-flow fractionation (PFF), dean-flow fractionation (DFF) and so on [48,49,50]. In the case of DLD, particles are separated based on a critical diameter (*D_c_*) associated with (1) distance between repeating patterns inside the channel, (2) the number of rows with repeated patterns, and (3) a tilted angle of the patterns along the column [48]. If the size of particles (*r_p_*) are smaller than *D_c_*, namely *r_p_* > *D_c_*, particles are contained in the 1^st^ flow streamline closest to the patterns and moved. On the other hand, if *r_p_* > *D_c_*, particles move out of the 1^st^ flow streamline and only move in a straight line. These different movements cause particles to be classified by size. Similar to DLD, PFF also separates particles by size, but uses a narrow channel structure called a pinched segment, not a repetitive microstructure inside the channel, to separate particles [49]. DFF separates particles using secondary flows, which are formed when primary flows rapidly into a spiral microfluidic channel [50]. 

### 3.8. Optical Trapping Force

Because light transmits momentum in proportion to the direction of energy and propagation, the momentum of light changes as the direction of light changes by reflection or refraction. This change in the momentum of light exerts a force on the particles by law of conservation of momentum, causing the particle to be trapped or separated in certain areas. Generally, a highly focused laser beam is widely used to capture particles effectively because the focused laser beam generates a strong field gradient at the center of the laser beam along the Gaussian beam waist. By the field gradient, the momentum of light changes and a force is generated, which attracts or disperses dielectric particles into the beam area [51]. The force traps or emits particle at the center of the laser beam and can be calculated taking into account the stiffness of the trap and distance from the center of the laser beam to the particle [52].

## 4. Novelty of DEP Force for Convergence of Various Forces

Among the various forces introduced in Section 4, DEP force causes two types of forces, namely p-DEP and n-DEP force, through simple frequency control, allowing biomolecules to be separated into different regions. In addition, the DEP force has a high biomolecular compatibility compared to EP and ETF force, which require relatively strong electric field and high temperature, respectively. Furthermore, unlike optical trapping force that requires large-scale optical systems and magnetic force that needs a labeling process for magnetic materials, DEP force has high simplicity and compatibility [53]. Such competitiveness of DEP force has led many researchers to develop methods for separating the biomolecules using single DEP or complex DEP force combined with other forces. In this section, various separating and detecting methods of biomolecules with DEP force were introduced by categorizing into four groups: using single DEP, multi DEP, dual DEP, and complex DEP force.

### 4.1. Single DEP Force

**Figure 1:**
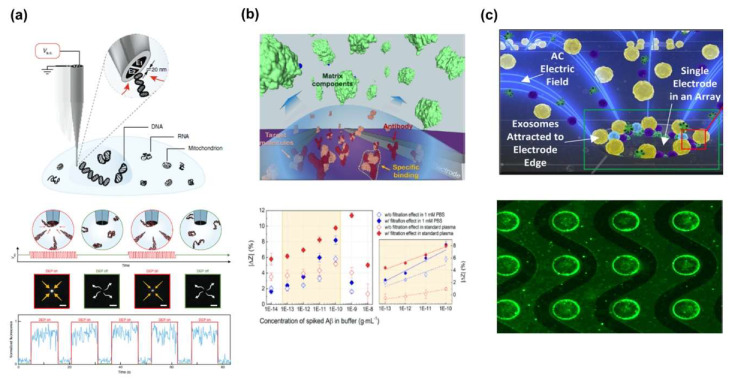
Dielectrophoretic (DEP) force-based separation and detection of the various biomolecules: (**a**) DNA separation and analysis [55]. Copyright (2018) Springer. (**b**) Aβ separation and detection in Interdigitated micro electrode (IME) sensors [57]. Copyright (2019) Elsevier. (**c**) Exosomes separation in micro-hole array [58]. Copyright (2017) American Chemical Society.

Initially, most of studies used only a single DEP force to separate and detect various biomolecules. Wu et al. presented separating cell organelles like nucleus (7–10 μm), mitochondria (0.1–10 μm) and lysosome (0.1–1.2 μm) from a whole cell by DEP. This separating method has a main advantage of analyzing single cells [54]. Nadappuram et al. showed a trapping and separating of 10 kbp DNA by applying DEP force between the tips of nanoscale tweezers with tens of nanometers of pore electrodes (Figure 1a) [55]. Aside from trapping single molecules, they also extracted nucleic acids for gene expression analysis from living cells without affecting their viability then trapped and extracted a single mitochondrion from the axon of mouse primary hippocampal neurons. Furthermore, DNA trapping by DEP force is represented with fluorescence intensity change by switching DEP force on or off. Oh et al. also separated, carried and released the DNA by applying the p-DEP force between the interdigitated microelectrode (IMEs), and then, moving the tweezer between the IMEs [56]. Kim et al. separated a target protein, amyloid beta (Aβ), from plasma composed of various plasma proteins using n-DEP force (Figure 1b) [57]. By n-DEP force, small Aβ was located in the detection region between IMEs, while large plasma proteins were pushed out of the detection region. 

In addition, the graph indicates that with the target protein separation applying n-DEP, filtration effect appeared differently with two types of buffer. Ibsen et al. also demonstrated a new method for exosome separating nanoparticles by using DEP force. They fabricated a device that had 400 microelectrodes trapped and detected the exosome by using DEP force at the edge of the microelectrodes. A fluorescence image verified that the exosome concentration comes to place at the edges of electrode (Figure 1c) [58]. These results showed DEP force can be used for separating and detecting various biomolecules. 

### 4.2. Multi DEP Force

One of the most attractive advantages of DEP force is that it can easily adjust the direction of force—p-DEP and n-DEP force—by controlling the alternating current (AC) frequency. Therefore, various studies have been reported to separate and detect biomaterials by simultaneously applying p-DEP and n-DEP force, namely multi DEP force, in a single bio-sensing platform or single microfluidic channel.

**Figure 2:**
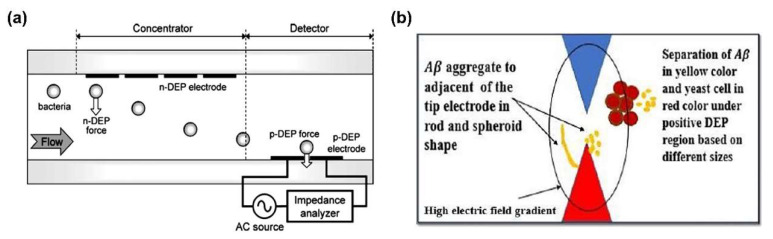
Separation of biomolecules by multiple DEP force combined with n-DEP and p-DEP force: (**a**) Bacteria separation in the Dielectrophoretic impedance measurement (DEPIM) fluid system [60]. Copyright (2013) Elsevier. (**b**) Aβ separation from yeast cell in microtip electrodes [62].

Lewpiriyawong et al. demonstrated continuous separation of yeast cells and bacterial cells from various particles as well as live yeast cells from dead yeast cells with high efficiency [59]. To secure high cell separation efficiency of approximately 97%, two types of DEP forces were used; p-DEP force was used to attract the live cells to the AgPDMS electrode, while n-DEP force was utilized to repulse the dead cells from the electrode. Hamada et al. also separated and detected the bacteria using n-DEP and p-DEP forces simultaneously. By n-DEP force, bacteria were pushed down in the cross section of the channel, and then, detected at the electrode where p-DEP force occurs. Dielectrophoretic impedance measurement (DEPIM) fluid system, utilizing DEP force, is a simple and rapid bacteria detection technique without any reagents. DEPIM fluid system containcontains n-DEP based bacterial concentrator, therefore, cells are affect n-DEP force to move toward to bottom of the DEPIM fluid channel then efficiently trapped at p-DEP microelectrode (Figure 2a) [60]. Similar to the above, Alazzam et al. separated breast cancer cells, MDA-MB-231, from cell mixture by attracting cancer cells with p-DEP and pushing blood cells with n-DEP [61]. Multi DEP force could be applied to separate protein; Ahdal et al. considered differences in CM factors between Aβ and yeast cells, causing Aβ to experience p-DEP force and cells to experience n-DEP force at the same AC frequency (Figure 2b) [62]. As a result, Aβ and yeast cells were successfully separated in the micro-fluidic channel.

### 4.3. Combination of DEP Force and Other Forces

As described above, the DEP force can be used in combination with other forces such as electrokinetic, magnetic, hydrodynamic, acoustic and optical trapping force, thereby, improving the accuracy and efficiency of biomolecule separation and detection. This chapter introduced various studies that combine the DEP and various forces to separate and detect various biomaterials.

When an AC voltage with a DC offset is applied to the electrodes, DC electromagnetic force and DEP force simultaneously occur on the same electrodes. Due to this simple mechanism, from the initial, many researchers have widely used dual force combining DEP and DC electrokinetic force for effective separation and detection of biomolecules.

Chang et al. used combined force of EP and DEP, analyzed a single DNA by sorting and stretching them at the nanopore [63]. By moving the DNA with the DEP force and stretching them with EP force, linearly stretched λ-DNA was observed through a fluorescence image. Rohani et al. utilized the AC fields with DC-offset to separate the prostate specific antigen (PSA) with anti-mouse immunoglobulin antibodies with the DEP and EP force in the nano-slit channel [64]. By using the combined force, they demonstrated the false positives can be eliminated, usually obtained with >10^−4^ folds higher levels of interfering anti-mouse IgG antibodies. Additionally, the dual force combining DEP and DC electrokinetic force was used to separate cells. Wu et al. used an AC voltage along with a DC bias voltage to generate traveling wave (TW) force and DEP force simultaneously and separated the yeast cells from polystyrene beads within 5 min [65]. Applied DC voltage and frequency were 12 V and 500 kHz respectively, and as a result, most of polystyrene microbeads travelled toward the center of the electrode array while the yeast cell almost was separated at the electrode edges by p-DEP force.

**Figure 3:**
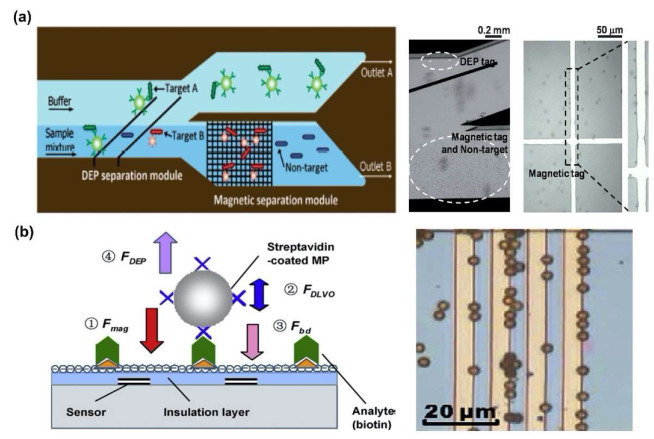
Magnetophoretic force combined dual DEP force-based biomolecules separation: (**a**) Two types bacteria cell separation in dielectrophoretic-magnetic activated bacteria sorter (iDMACS) chip [66]. Copyright (2009) Royal Society of Chemistry. (**b**) Separation of streptavidin using streptavidin-coated magnetic particles [67] Copyright (2009) Elsevier.

Although magnetophoretic force generally requires permeable particles to induce the magnetophoretic response of biomolecules, it is insensitive to the properties of the buffer, especially the conductivity of the buffer. Therefore, magnetophoretic force has been used in conjunction with DEP force for biomolecules separation and detection. Kim et al. fabricated the dielectrophoretic-magnetic activated bacteria sorter (iDMACS) combining DEP and magnetic force to separate the two types of bacterial cells with high efficiency (Figure 3a) [66]. To separate and detect the cells using DEP and magnetophoretic force simultaneously, a magnetic tag was attached to the target cell and a mixture containing the target cell and the non-target cell was injected into the channel where the electrode for DEP was formed. The magnetophoretic force was also used in conjunction with the DEP force to separate protein. Liu et al. demonstrated a method to separate the streptavidin using DEP force and magnetic particles, which was coated by streptavidin. To separate the streptavidin-coated magnetic particles, the magnetic particles were attracted using magnetophoretic force, and then, the non-specific protein was repelled using n-DEP force (Figure 3b) [67].

Hydrodynamic forces can be simply generated by structures of microfluidic channel without additional systems and electrode fabrication. For this reason, hydrodynamic force has been frequently used to separate and detect the biomolecules in combination with DEP force.

**Figure 4:**
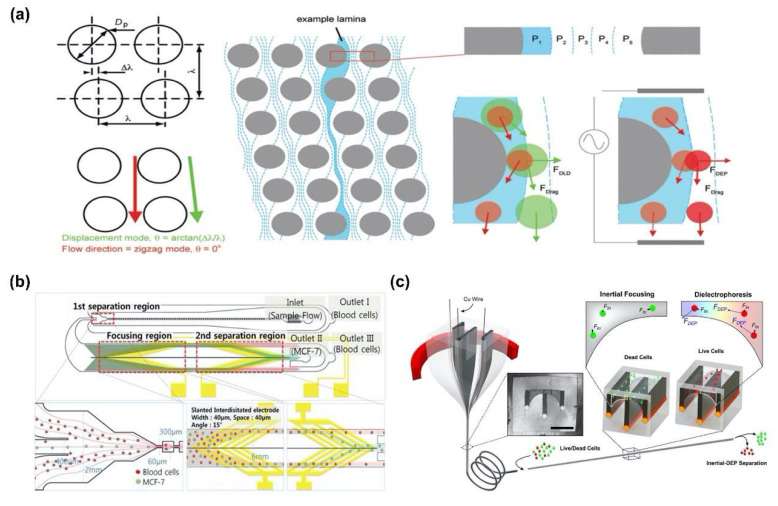
Biomolecules separation using hydrodynamic force combining dual DEP force: (**a**) Separation of bacterial artificial chromosomes and blood cell using deterministic lateral displacement (DLD) and DEP force [68]. Copyright (2009) Royal Society of Chemistry. (**b**) Multi-orifice flow fractionation (MOFF) and DEP force for continuous separation of breast cancer cells (MCF-7) from blood [69]. Copyright (2011) Royal Society of Chemistry. (**c**) Live cell separation from dead cell in microfluidic channel by inertia force and DEP force [70].

Beech et al. separated bacterial artificial chromosomes and blood cells by combining DEP force and DLD phenomenon (Figure 4a) [68]. They calculated the *D_c_* of the fluid channel with micropillars, and then, used the DEP force to control the position of the biomolecules in the region from the surface of pillars to the *D_c_*. As a result, the pathway of the moving biomolecules was changed by DEP force, namely biomolecules were separated depending on the size of the biomolecule with high efficiency. Moon et al. suggested continuous separation of breast cancer cells (MCF-7) from blood using multi-orifice flow fractionation (MOFF) force and DEP force (Figure 4b) [69]. To separate cancer cells, human blood samples were injected into a microfluidic channel with two separate regions; the 1st region was based on the MOFF force and the 2nd region used DEP force to separate and detect biomolecules. Consequently, efficiencies of about 99.24% and 94.23% have been demonstrated for the separation of red and white blood cells, respectively. In addition, Yuan et al. separated live cell and dead cell in microfluidic channel by using inertia force and DEP force [70]. To induce two types of forces, the fiber microfluidic channel was fabricated by controlling the cross-sectional shape of the channel and incorporating a conductive material along the channel surface. Thus, live and dead cells were affected by different intensity of inertia and DEP force and separated into different positions within the fiber with a high throughput of 100 μL/min (Figure 4c) [70].

**Figure 5:**
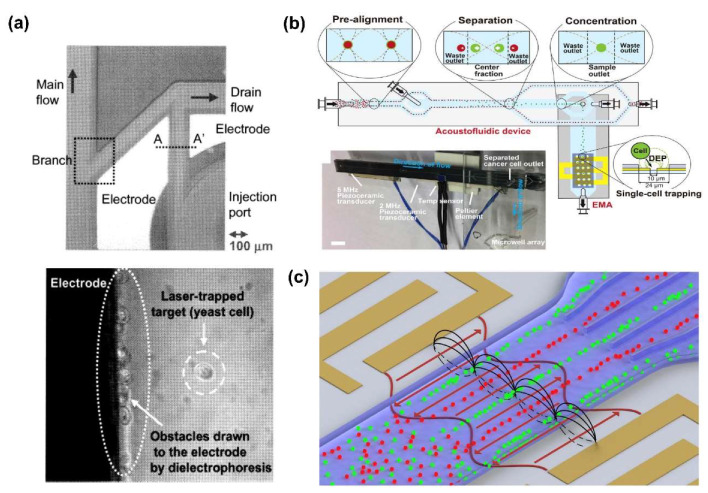
Biomolecules separation using dual DEP force combined with optical force or acoustophoresis (ACP) force: (**a**) Laser-trapping force combining dual DEP force to separate a living cell and microbe in microfluidic device [72]. (**b**) Separation and detection of circulating tumor cell (CTC) from peripheral venous blood by combining an ACP and DEP force [73]. (**c**) Separation of two types of cell by surface acoustic wave (SAW) and DEP [74].

DEP force was also used in conjunction with optical and acoustic force. Maruyama et al. combined DEP force and optical tweezer technology to concentrate and analyze low concentration of influenza virus [71]. The virus was effectively concentrated by n-DEP force on the electrodes, then analyzed by optical tweezer. Additionally, Arai et al. integrated the laser-trapping and DEP force to separate a single microorganism, such as a living cell and microbe in microfluidic device (Figure 5a) [72]. They captured the target microbe at the focal point of the laser and used DEP force to separate it from the excess microbe around the target. As a result, the target microbe was quickly separated within 20 s.

On the other hand, Antfolk et al. developed a microfluid device capable of simultaneous separation and detection of circulating tumor cell (CTC) from peripheral venous blood by combining an ACP and DEP force (Figure 5b) [73]. Smith et al. also separated non-viable human stromal cells using DEP based shear-horizontal surface acoustic waves (SAW) (Figure 5c) [74]. 

### 4.4. Complex Convergence of Forces for Various Functions

As a more advanced method, various attempts have been made to separate and detect the biomolecules using complex force that combines DEP force with two or more forces recently. The complex forces facilitated the separation and detection of biomolecules with high sensitivity and accuracy. In this section, we explained various results of separation and detecting cells, protein and exosomes with high efficiency and sensitivity using complex force.

**Figure 6:**
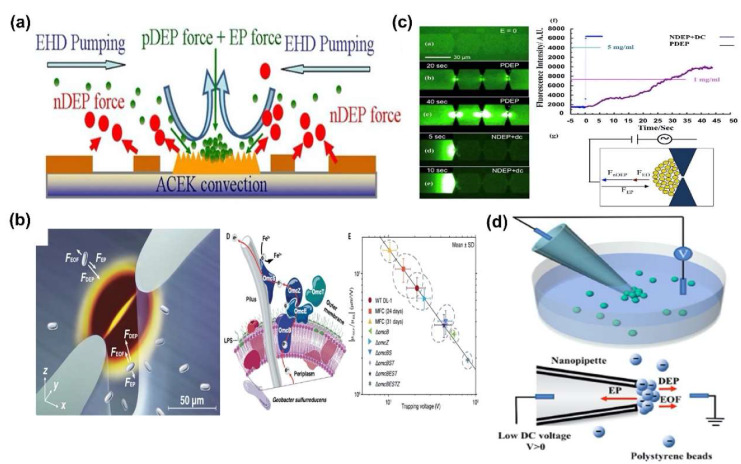
Separating and analyzing of various biomolecules using complex DEP force: (**a**) Bacteria and blood cells separation in microfluidic chip using the DEP, electrohydrodynamics (EHD) and electrophoretic (EP) force [75]. (**b**) Separating and analyzing of microbial cells using a complex DEP force combined with EP and electroosmotic flow (EOF) force [76]. (**c**) Rapid separation and detection of protein and exosomes using EOF, EP, and DEP force [77]. Copyright (2017) Elsevier. (**d**) Exosome separation from the plasma of the healthy donors and liposome using complex DEP force combined with EP and EOF force [40].

Cheng et al. selectively separated the bacteria cells from human blood cells, such as red blood cells and white blood cells by utilizing DEP, EP and electrohydrodynamics (EHD) forces simultaneously (Figure 6a) [75]. They controlled the AC frequency range to apply n-DEP and EHD force to bacterial cells and p-DEP force to blood cells simultaneously. p-DEP, n-DEP and EP force can be produced by AC electric field induced electrokinetic force (ACEK) convection to separate and concentrate the pathogens in the detection region. Subsequently, they established the optimal intensity of three forces for selectively separating the bacterial cells from the blood by concentrating the bacterial cells in the sensing region and excluding the blood cells from the region. In the optimal AC voltage and frequency range, consequently, bacteria cells with size of 0.5–2 μm were efficiently separated from the blood cells with sizes of 6–8 μm within 5 min, of which separation efficiency was approximately 40–50%. Similarly, Wang et al. separated and detected microbial cells using a complex force based on DEP force to analyze microbial cell envelope polarization and electrochemical activity (Figure 6b) [76]. To separate the microbial cells, they considered the electrokinetic mobility, stroke’s drag force, and DEP force after assuming that the intensity of the Brownian motion and the effect of cellular motility were smaller than DEP force. The complex force was induced the in three-dimensional (3D) microfluidic channel with a cross-sectional area which was 100 times smaller than vicinity of the constriction. Then, the microbial cells were concentrated near the constriction when the DEP force was balanced against drag force, which was induced by the EOF and EP force in the channel. As a result, the microbial cells were trapped in small cell culture volumes for a short amount of time and surface polarizabilities for wild-type strains and cytochrome deletion mutants of two model extracellular electron transfer (EET) microbes, *Geobacter sulfurreducens* and *Shewanella oneidensis*, were analyzed. In addition, surface polarizabilities for *Escherichia coli* strains heterologous expressing Shewanella oneidensis EET pathways were investigated in various growth conditions. Through the results, they demonstrated a correlation between bacterial EET and surface polarizability, which showed the complex DEP force could be utilized to analyze the properties of the cell.

In addition, the complex force allowed for the rapid separation and detection of protein and exosome. Modarres et al. showed an ultrafast protein enrichment method with complex force of n-DEP, EO and EP (Figure 6c) [77]. In order to apply the complex force, a nanofluid channel with slanted nanostructure was fabricated to make a molecular dam, and AC voltage with DC bias was applied to the channel. In the channel, the n-DEP and EO force acted as a repulsive potential that interfered with the flow of biomolecules, while the EP force acted as an attractive potential that caused molecular flow. Thus, biomolecules accumulated in the molecular dam under optimum conditions, where the difference between the combining force of the n-DEP, EP and EO force was minimized. In the optimum condition, IgG were enriched by the molecular damming effect faster than the trapping effect, to >10^5^-fold in 20 s. Additionally, Shi et al. initially separated 100 nm diameter of polystyrene bead, that is a similar size of exosome, by nanopipette and various forces. Furthermore, they successfully separated the exosome from the plasma of the healthy donors and liposome resuspended in the PBS solution by using the electrokinetic forces including EOF, EP and DEP force (Figure 6d) [40]. To separate the exosomes and liposomes, a nanopipette was fabricated and a positive biased DC voltage was applied across the pipette length. When the DC voltage bias was applied at the base of the pipette, the intensity of EP force maximized at near the pipette’s tip, while the EOF and DEP force were minimized near the tip. Thus, depending on the tendency of these forces, the three types of forces were balanced under certain conditions, and consequently, exosomes were trapped at the nanopipette’s tip. A strength of each force was calculated according to an intensity of applied voltage, ionic concentration of buffer, size of nanopipette and separation of the exosome was demonstrated by observing the fluorescence images as well as measuring the change of conductance. As a result, exosome and liposome were trapped and released at the tip of nanopipette within 100 s according to the polarity of applied voltage and zeta potential of the molecules. 

So far, we have introduced various examples of using single DEP force, multi DEP force and converged complex forces based on DEP force to perform various functions such as biomolecule separation, concentration and analysis. These examples mean not only the possibility of a single DEP force, but also the ability to expand to various areas through fusion with other forces. 

## 5. Conclusions

Separation of target biomolecules provide great possibility to detect the target biomolecules with high selectivity and sensitivity. Recently, applying complex force-based separation of biomolecules for more functions in microfluidics have achieved simple and high yield separations.

Furthermore, a lot of convergence of forces would lead more abilities of microfluidic chips as a lab-on-a-chip. In this review paper, DEP force was preponderantly introduced as a great candidate force to accomplish various functions in microfluidics with its novelty. The DEP force is a promising force to accomplish various functions in microfluidics with less processes. Therefore, there were many studies and cases applied DEP force with different kinds of forces.

In addition, unlike other forces, there were also many studies about applying only DEP force for separation or filtration of DNA, protein or exosome. The point also shows the novelty of DEP force. We expect more kinds of forces to be combined in a complex way in the future, resulting in improvement of technologies for detecting target biomolecules. Thus, the DEP force is also expected to always be the core in convergence of forces with its novelty.

## Figures and Tables

**Table 1:** The properties of biomolecules affecting various forces that separate the biomolecules.

Properties of Biomolecule	Definition
Permittivity	The dielectric permittivity of biomolecules can be defined as an ability of a substance to hold an electric charge. Depending on the permittivity, biomolecules are affected by the different types of DEP force, resulting in separation.
Surface charge	The surface charge of biomolecules results from ionization of carboxyl, phosphate or amino groups and ion adsorption from solutions. The surface charge of the biomolecules can be characterized by the zeta potential, which is the potential at the interface between the medium and the stationary layer of the biomolecule and is determined by the Smoluchowski formula as follows [37]: $$\zeta =-\frac{\mu \eta }{\varepsilon_{0}\varepsilon_{r}}$$ where, ζ represents zeta potential, μ is electrophoretic mobility, η is the viscosity of the electrolyte solution, $\varepsilon_{0}$ and $\varepsilon_{r}$ are the relative permittivity of the electrolyte solution and a vacuum, respectively. Thus, the intensity of zeta potential affects the biomolecule’s behavior during electrophoretic separation.
Compressibility	The compressibility is a measure of the relative volume change of a fluid or solid as a response to a pressure change, and the degree of compressibility has strong implications for its hydrodynamics. In particular, since propagation of sound depends on the compressibility between biomolecules and media, biomolecules are affected by the acoustophoretic force depending on the biomolecule’s compressibility.
Size	The size of a biomolecule is a biological property that affects most forces, such as DEP, EP and ETF. Exosomes, cells, and bacteria have a specific shape, but DNA, especially proteins, have a non-uniform shape, so it can be modeled as spherical via the Erickson equation as follows [38]: $R_{min}=0.066M^{\frac{1}{3}}$ where R and M indicate the radius and molecular weight of biomolecules, respectively.
